# Mr. Wallace on a Fungous Eruption—Dr. Elliotson on Diarrhœa

**Published:** 1828-04-01

**Authors:** 


### XIII.
### MEDICO-CHIRURGICAL TRANSACTIONS.

1. *Remarkable Fungous Eruption curable by Mercury.* By Mr. WALLACE.

THE unsightly disease which forms the subject of this communication appears to be much more frequent in the Sister Island than here. Mr. Wallace, who is attached to an institution for cutaneous diseases in Dublin, ventures to give it the name of morula, from morus, a mulberry. This term marks its most prominent feature—" a fungus of a rounded and granulated form." Although its appearances are regular and peculiar, it has not previously been described. Its growth is somewhat similar to that of the yaws; but there are differences in other respects, which forbid its classification with that disease. Our author has not had occasion to see the eruption, except among hospital patients—and it is curious that the majority of the individuals were either males or females, who got their livelihood by traffic in old clothes, rags, and similar merchandize. It has frequently been observed, however, by others among the peasantry of Ireland. Mr. W. does not think it has any necessary connexion with syphilis, nor pruriginous affections—nor, in short, with any other disease. On some occasions, there was reason to suspect that it was propagated by immediate contact—but generally it appeared void of contagious character. There is scarcely a part of the body, except the palms of the hands and soles of the feet, upon which Mr. W. has not observed these fungous eruptions.

"They uniformly commence, as far as my observation goes, in minute pimples, which become quickly covered on their apex by a very small scab, upon the removal of which may be observed the germ of the future fungus, consisting of a single granulation, and so minute as to require for its discovery the assistance of a lens. At this period the spot is itchy, and is surrounded by a slight erythematous redness. Its size gradually but progressively increases. In the course of some days, a scab of several lines in breadth, of a brownish yellow colour, and considerably elevated, will be found to cover a fungus of a rounded figure and granulated surface, of a yellowish red colour, sore to the touch, and surrounded by a slight livid redness. The size to which these spots are capable of increasing as fungi is, I believe, limited. I have never observed them larger than about one inch and a quarter in diameter, and I have generally remarked that when the spot acquired about an inch in diameter, the action of the vessels of the part changed, and the fungus becoming absorbed, an ulcer was produced. If credit could be given to the observation and reports of the patients themselves, it would appear that many of the fungi, upon arriving at a certain magnitude, shrink and fade away; but whether a fungus, after it has been once decidedly formed, ever disappears, except by the formation of an ulcer, without the interference of art, is a point upon which I cannot speak decidedly from my own observation."

It is very remarkable that the part on which these fungi have been situated, possesses the power of healing, without the formation of any permanent cicatrix —demonstrating that the fungi grow from the *surface* of the cutis, and that the texture of this covering is not permanently injured. The number of fungi in individuals, varies from one to fifty. The THERAPŒIA of the subject will clearly appear in the following extract.

"In the whole catalogue of maladies which are capable of being cured or relieved by mercury, I do not know any which exhibits the value of this mineral more remarkably than the disease in question. I believe it matters nought whether this valuable agent be employed internally or externally. If internally, whether it be used as an oxide or salt; if externally, whether it be employed in the form of an unguent, vapour, or fluid, for the disease immediately shrinks, *as soon as the slightest mercurial action in the system is manifested.* I generally retain the patients under care five or six weeks, and use the remedy to such an extent as to cause its gentle but marked influence on the system; and the form in which I employ it is varied according to the peculiar circumstances or convenience of each case."

Three cases and an expressive plate are given by Mr. Wallace, in illustration of the eruption above described.

### 2. *Sulphate of Copper in Chronic Diarrhœa.* By Dr. ELLIOTSON.

IN a former number of this Journal, (No. 13, p. 156,) we alluded to Dr. Elliotson's practice of exhibiting sulphate of copper in chronic bowel-complaints, and his paper is now before the profession in the Medico-Chirurgical Transactions.

Our intelligent author seems to have been led to the practice in question, by the case of a man who was admitted into Guy's Hospital in 1824, labouring under diarrhœa of three months standing. The abdomen being tender on pressure, the pulse quick, and the skin hot, the motions yellowish and watery, he was ordered a blister, chalk mixture, and half a grain of opium, night and morning. The diarrhœa continued, and then various astringents with opium were tried—but still the diarrhœa persisted as profuse as ever, and the man appeared to be sinking. Dr. E. now learnt from a pupil, that the sulphate of copper had been successful in some cases of this kind, and he immediately gave it a trial, prescribing half a grain twice a day, with two grains of opium in the 24 hours. In a few days the disease was less severe—and in eleven days from the commencement of this plan, the patient was so far relieved, that the opium was considerably diminished, and the sulphate increased. In a fortnight more the man was well.

Dr. Elliotson next alludes to the disease noticed by Dr. Baillie, as "not very well known, and almost always fatal." He describes it as occurring oftener in males than in females, and generally in those who have resided long in hot climates. He says, the stools are very copious and numerous, pale, like a mixture of water and lime, frothy, and often of a sour smell. Even when they acquire the consistence of pudding, they are still pale—and if they become figured and dark, the colour is rarely that of healthy motions, and they soon become white and frothy again. The body emaciates—the countenance is sallow—and the constitution is ultimately destroyed. A man, a sailor, who had resided in hot climates, and whose complaint tallied with that described by Dr. Baillie, next came into the hospital, and the treatment above-mentioned was employed. In four days the dose of sulphate was increased to one grain twice a day. In a few days the stools were reduced to two or three in the 24 hours. In less than a month the motions assumed a deep healthy yellow. A relapse, however, occurred, and the dose was augmented. The patient was ultimately discharged cured. Several other cases are detailed, but we deem it unnecessary to dwell upon them. Dr. E. considers the medicine in question as superior to every other astringent in chronic diarrhœa—and thinks it will cure the disease more quickly than any other—and often when all others fail. Three grains, thrice a day, is the largest quantity he has given. Some patients took it in this quantity three or four months, without any inconvenience. But, as it has a tendency to produce vomiting and griping, Dr. Elliotson *always* combined it with opium, except for about a week in the first case, and towards the decline of the disease. Our author concludes that it was principally owing to the sulphate that his patients recovered, since the opium had been previously used in various forms, without success.

Granting the utility of the remedy here introduced, it must be remembered that the diet, the quietude, and the regularity of an hospital, are powerful means of curing bowel-complaints. We venture to affirm that, if one half of any number of patients, labouring under bowel-complaints, were confined to their beds, and fed upon farinaceous food, without a particle of medicine, they would sooner get well than the other half allowed to go about the wards, and treated with the sulphate of copper, or any other medicine or combination of medicines in the Pharmacopœia. We regard the horizontal posture, warmth, and farinaceous food, as the basis of all treatment in bowel-complaints—and superior to all other medicines, where this regimen is not enjoined. In respect to the modus agendi, we adhere to our former opinion, that it is not merely by its astringency that it checks diarrhœa. It is to be recollected that the mucous membrane of the intestines in this disease, is morbidly irritable—and the sulphate of copper, very probably, acts internally as it does externally, by diminishing this inordinate sensibility of the mucous surface. It is chiefly in this way that opium is useful.

In a postcript to this valuable paper, Dr. Elliotson alludes to a former communication on large doses of the subcarbonate of iron—as also on prussic acid, pulvis antimonialis, and sulphate of quinine. With this last medicine Dr. E. has cured nearly 150 cases of intermittent fever—many of them combined with inflammation of the great organs of the body, requiring venesection—some of them combined with dropsy—some with chronic disease of lungs or liver—"*but every one was cured.*" He has never seen it augment any inflammation that might be present, or interfere with antiphlogistic measures; consequently he has given it under all circumstances, "and simultaneously adopted any other measures that might be demanded by other symptoms." How will these facts quadrate with Mr. Lawrence's observations on bark, in his celebrated speech on erysipelas?

Dr. E's further experience with carbonate of iron enables him to state that he has treated nine cases of chorea with this remedy, (generally in doses of three or four drachms,) and did not once fail. The time usually required varied from six to ten weeks. Dr. E. has treated a genuine case of traumatic tetanus with it, in doses of half an ounce every two hours.

Dr. E. is anxious also to make a report on *acupuncture*, which he has employed very extensively, both in private practice and at St. Thomas's Hospital. His experience perfectly coincides with that of Mr. Churchill;—namely, that it is chiefly useful in the rheumatism of fleshy parts—rheumatalgia, and the more so, as the disease is less inflammatory. When, indeed, the parts are hot, or the pain is increased by heat, the remedy is generally useless, and cannot supply the place of antiphlogistic measures. He has found that one needle allowed to remain an hour or two in a part, is more efficient than several used but for a few minutes. "The effects are often magical." The pain sometimes ceases while the needle is in the flesh; but generally three or four introductions are necessary. Of 42 cases, taken in succession, from the hospital books, 30 were cured—the other twelve were not proper subjects. It is occasionally a good mode of letting off the fluid of anasarca; but the needle should not be pushed deep. "Neither is its use always attended with *safety*, as in rheumatism."

Dr. Elliotson, who is always practical, and rarely indulges in speculation, deserves the thanks of his cotemporaries for the candour with which he details the results of his experience in a public institution. Would that his brethren of public hospitals would follow his example!

### XIV.

*Lectures on the Intellectual Composition of Man. (Delivered at the London Medical Society.)* By DR. HASLAM.

